# Tailored postoperative treatment of prostate cancer: final results of a phase I/II trial

**DOI:** 10.1038/s41391-018-0064-7

**Published:** 2018-07-23

**Authors:** Giovanna Mantini, Giambattista Siepe, Anna Rita Alitto, Milly Buwenge, Nam P. Nguyen, Andrea Farioli, Riccardo Schiavina, Francesco Catucci, Francesco Deodato, Bruno Fionda, Vincenzo Frascino, Gabriella Macchia, Maria Ntreta, Gilbert D. A. Padula, Alessandra Arcelli, Silvia Cammelli, Giuseppe Zanirato Rambaldi, Savino Cilla, Vincenzo Valentini, Alessio G. Morganti

**Affiliations:** 1 Radiation Oncology Department Gemelli-ART, Fondazione Policlinico Agostino Gemelli, Università Cattolica del S. Cuore, 00168 Rome, Italy; 2 Radiation Oncology Center, Department of Experimental, Diagnostic and Specialty Medicine – DIMES, University of Bologna, S. Orsola-Malpighi Hospital, 40138 Bologna, Italy; 3 Department of Radiation Oncology, Howard University College of Medicine, Washington DC, 20059 USA; 4 Departments of Medical and Surgical Sciences - DIMEC, University of Bologna, S. Orsola-Malpighi Hospital, Bologna, 40138 Italy; 5 Department of Urology, University of Bologna, Bologna, S. Orsola-Malpighi, Bologna, 40138 Italy; 6 Radiotherapy Unit, Fondazione di Ricerca e Cura “Giovanni Paolo II”, Catholic University of Sacred Heart, Campobasso, 86100 Italy; 7 Cancer Research Consortium of West Michigan (CRCWM), Michigan State University, East Lansing, 48824 USA; 8 Medical Physic Unit, Fondazione di Ricerca e Cura “Giovanni Paolo II”, Catholic University of Sacred Heart, Campobasso, 86100 Italy

## Abstract

**Backgroud:**

The European Organization for Research and Treatment of Cancer (EORTC) trial 22,911 reported 74% 5-year biochemical disease-free survival (bDFS) in patients with prostate carcinoma treated with radical prostatectomy (RP) followed by postoperative radiotherapy (RT). This study aimed to improve these outcomes by using a combined-intensified-modulated-adjuvant treatment, including RT and hormone therapy (HT) after RP.

**Materials and methods:**

This phase I/II trial treatment was designed to improve 5-year bDFS from ~ 75 to 90%. Patients were consecutively enrolled using the following inclusion criteria: age < 80 years, histological diagnosis of prostate adenocarcinoma without known metastases, stage pT2-4N0-1, and Eastern Cooperative Oncology Group performance status of 0–2. All patients had at least one of these pathologic features: capsular perforation, positive surgical margins, seminal vesicle invasion, and pelvic lymph nodes involvement. A minimum dose of 64.8 Gy to the tumor bed was delivered in all patients. Depending on tumor characteristics at diagnosis, patients received a higher dose (70.2 Gy; 85.4%) and/or prophylactic pelvic lymph nodes irradiation (57.7%) and/or HT (69.1%). Biochemical relapse was defined as two consecutive rising prostate-specific antigen (PSA) values > 0.2 ng/ml.

**Results:**

A total of 123 patients were enrolled in the study and completed the scheduled treatment. Median preoperative and postoperative PSA were: 8.8 and 0.06 ng/mL, respectively. The percentages of patients with pathologically involved nodes and positive resection margins were: 14.6% and 58.5%, respectively. With a median follow-up of 67 months (range: 37–120 months), the actuarial 5-year bDFS, local control, metastasis-free survival, and overall survival (OS) were: 92.9%, 98.7%, 96.1%, and 95.1%, respectively.

**Conclusion:**

A higher 5-year bDFS (92.9%) was recorded compared to studies based on standard adjuvant RT, even though patients with nodal disease and detectable postoperative PSA were enrolled. Clinical end points, as long-term disease-free survival and OS, will require further assessments. (ClinicalTrials.gov: NCT03169933)

## Introduction

Despite a progressive decrease in mortality rates, prostate cancer (PCa) still represents the third cause of cancer-related death in Europe [1]. Radical prostatectomy (RP) is an effective treatment for localized PCa. Nevertheless, a significant percentage of patients (15–60%) develop recurrences after surgery and therefore require salvage radiotherapy (RT) [2–8]. Several randomized studies have demonstrated the benefit of adjuvant RT after RP in selected patients at high risk of failures [3–5].

An improvement in biochemical disease-free survival (bDFS) was first reported by EORTC 22,911 trial in 2005 [9]. The rate of biochemical failure remained significant (25% after 5 years). Based on the results of that study, we hypothesized that RT dose escalation to tumor bed, pelvic lymph node irradiation (PNI) in selected patients with higher risk of regional failures, and adjuvant hormone therapy (HT) for those with a higher risk of distant metastases could further reduce the recurrence rates.

In fact, with a dose higher than 60 Gy on prostatic and seminal vesicles bed, an improved bDFS was previously recorded [10]. In addition, patients at high risk of local failures such as those with positive surgical margins and/or perineural invasion may benefit from further increased doses (up to 70.2 Gy), to minimize recurrence rates [11, 12]. PNI may also reduce regional recurrences in selected patients at high risk for nodal involvement [13]. In fact, some studies have demonstrated an improved bDFS after prophylactic nodal irradiation also in post-prostatectomy setting [14–16]. Furthermore, improved bDFS in patients with a high risk of recurrence after RP with the combination of adjuvant HT and RT have been reported [17, 18].

Thus, considering all these factors, we defined combined-intensified-modulated-adjuvant (CIMA) treatment, as a new modality that may potentially improve patients’ outcome, by selectively using RT dose escalation, PNI, and HT based on individual patient risks after RP. The feasibility of CIMA has been previously tested in a preliminary analysis [19]. We now report the long-term outcomes of this study.

## Materials and methods

### Study objectives

The primary trial objective was to test the possibility to improve 5-year bDFS from 75 to 90%, as calculated from date of surgery to biochemical relapse. Biochemical relapse was defined as two consecutively rising prostate-specific antigen (PSA) values and a PSA level > 0.2 ng/mL. Secondary end points included early and delayed treatment-related side-effects, local control (LC), and metastasis-free survival (MFS). Patients without the events of interest were censored at their last contact date (last PSA assessment).

### Study design

A phase I/II trial was planned. A previously published randomized study [9] showed 75% 5-year bDFS in patients treated with standard adjuvant RT (dose: 60 Gy, no PNI, no HT). Considering 90% as the true success rate for our experimental cohort, 100 experimental patients were needed to reject the null hypothesis, that the success rates for CIMA and historical patients are equal with probability (power) 0.8. The 0.05 type I error probability is associated with the test of this null hypothesis. An uncorrected *χ*^2^ statistic was used to evaluate this null hypothesis. Some over-recruitment was planned to compensate for 20% drop-out after enrollment.

### Inclusion criteria

Patients < 80 years, with resected non-metastatic PCa not previously treated with RT, HT, or chemotherapy (CT) and free from surgical complications were enrolled. Furthermore, patients had at least one of the following risk factors: extracapsular extension, and/or positive surgical margins, and/or seminal vesicle infiltration, and/or regional lymph nodes invasion. Undetectable postoperative PSA was not considered as an inclusion criterion for the study. We used the International Union Against Cancer criteria [20] to define tumor stages. All patients were evaluated by PSA (preoperative and postoperative), abdominal and pelvic CT or MRI, and bone scans prior to enrollment. Patients with distant metastases, extra-pelvic lymphadenopathies, and macroscopic residual disease were excluded. All patients had an Eastern Cooperative Oncology Group (ECOG) performance status between 0 and 2, and adequate bone marrow function (hemoglobin concentration > 8 g/dl, white blood cell count > 3000/mm³, platelet count > 75,000/mm³).

### Therapy

#### Radiotherapy

The details of the three-dimensional (3D) conformal RT technique were described in our previous report [19]. Prior to the planning scans, all patients were given detailed instructions about positioning (supine) and bladder and bowel filling to attain reproducibility during simulation and throughout RT administration. Based on Radiation Therapy Oncology Group (RTOG) guidelines for the definition of the clinical target volume in postoperative conformal RT, we defined two CTVs: CTV1 and CTV2. CTV1 included the prostate and seminal vesicles bed, whereas CTV2 included obturator, internal iliac, external iliac, and presacral (above S2–S3) nodes.

All patients received postoperative RT with set-up evaluation and correction if needed (using Electronic Portal Imaging Device) daily, 5 days a week. We used the International Commission of Radiation Unit 62 guidelines [21] for dose specification and in consideration of tumor characteristics (Table 1), doses were prescribed accordingly: (i) PNI (45 Gy; 1.8 Gy/fraction) plus boost to the prostate bed (19.8–25.2 Gy; 1.8 Gy/fraction; total dose: 64.8–70.2 Gy) or (ii) exclusive prostate bed irradiation (64.8–70.2 Gy; 1.8 Gy/fraction).

**Table 1:** Prescribed treatment based on patients/tumor characteristics

Treatment modulation	Patients/tumor characteristics
Higher dose (70.2 Gy) to the tumor bed	Positive surgical margins and/or perineural infiltration and/or postoperative PSA> 0.2 ng / mL
PNI	pN1 and/or lymph node risk > 15% ^a^ and < 10 resected nodes and/or Gleason score > 7
Short-term (6 months) HT	pT > 2 and/or Gleason score = 7
Long-term (24 months) HT	pN1 and/or preoperative PSA > 20 ng/mL and/or Gleason score > 7

^a^Based on Roach 3rd M (Roach 1993); HT: hormone therapy; PNI: prophylactic nodal irradiation; PSA: prostate-specific antigen.

#### Hormone therapy

Table 1 reports HT prescriptions. At commencement of adjuvant RT, patients started either LH-RH analog (leuprorelin, 11.25 mg  every 3 months, intramuscularly) or antiandrogen agent (bicalutamide, 150 mg daily per os). Based on risk factors (T stage and Gleason score (GS), to the patients were prescribed short time (6 months) or long time (24 months) HT.

### Statistical analysis

A descriptive analysis of the sample was carried out using mean and standard deviation for continuous variables, whereas absolute and relative frequencies for qualitative ones. Patients were monitored weekly during RT. Acute side-effects were scored according to the RTOG scale [22]. Late complications were assessed with the Late Radiation Morbidity Scoring Scheme of the RTOG/European Organization for Research and Treatment of Cancer (EORTC) [22]. Clinical assessment included serum PSA level and digital rectal exam every 3 months for the first 2 years, biannually in 3rd, 4th, and 5th years, and annually thereafter. Additional studies such as bone scans or CT/MRI were requested if there were clinical suspicions of recurrences or increasing PSA levels. Analyzed variables were: age at diagnosis (≤ 65 vs. > 65), pathological evaluation on the extent of the primary tumor (pT2 vs. pT3–4), pathological evaluation of regional lymph nodes (pN0 vs. pN1 vs. pNx), margin status (R0 vs. R1), perineural infiltration (no vs. yes), PSA pre-surgery (≤ 10 ng/mL vs. > 10 ng/mL), PSA post surgery (≤ 0.2 ng/mL vs. > 0.2 ng/mL), histopathologic grade (GS ≤ 7 vs. GS 8–10), lymphadenectomy (no vs. yes), surgical bed dose (64.8 vs. 70.2 Gy), PNI (no vs. yes), HT (no vs. yes), type of HT (antiandrogen vs. LH-RH analog), and duration of HT (short-term: 6 months vs. long time: 24 months). We evaluated the impact of these factors on bDFS. Furthermore, analysis of bDFS, LC, MFS, and overall survival (OS) was performed. Survival curves were calculated with the Kaplan–Meier product-limit method and stratifications for selected prognostic factors were assessed for statistical significance using the log-rank test statistic [23, 24]. Statistical analysis was carried out using SYSTAT, version 11.0 (SPSS, Chicago, IL). A two-sided *p* value of 0.05 was considered statistically significant.

### Ethical issues

All patients consented to treatment and provided a written informed consent to enrollment in the clinical trial. Our institutional review board approved the study. Patients were enrolled from 2004 to 2009. The study is registered in an international public registry (ClinicalTrials.gov Identifier: NCT03169933).

## Results

**Fig. 1:**
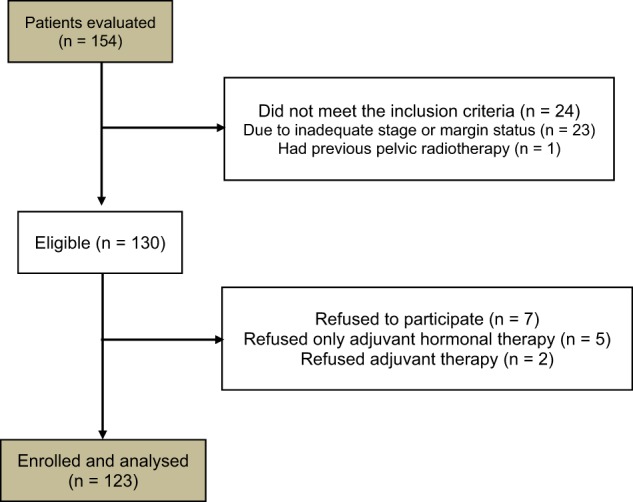
Consolidated Standards of Reporting Trials (CONSORT) diagram

**Fig. 2:**
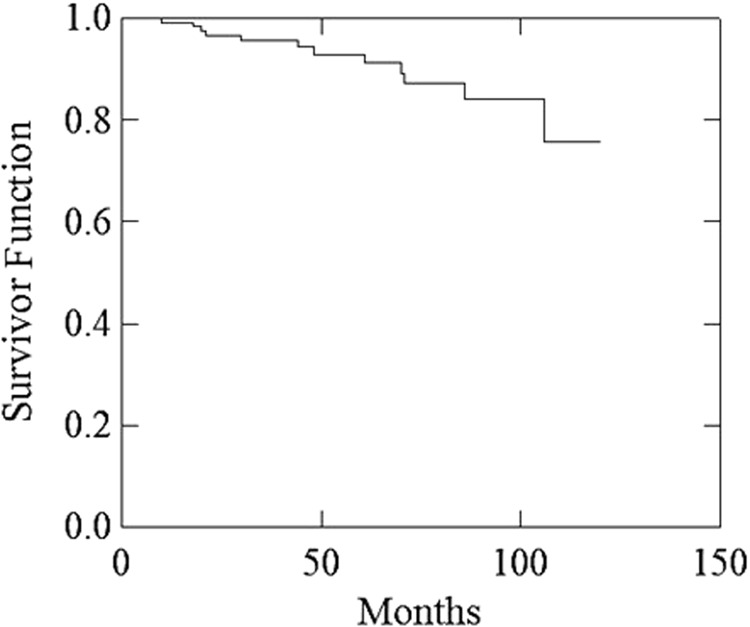
actuarial biochemical progression-free survival

**Fig. 3:**
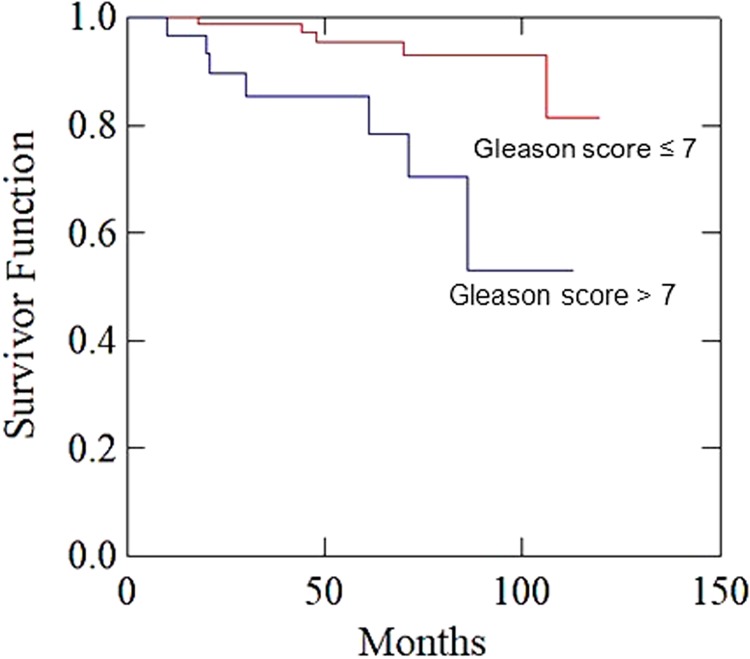
impact of Gleason Score on biochemical progression-free survival

Median follow-up was 67 months (range 37–120 months). Figure 1 illustrates the Consolidated Standards of Reporting Trials (CONSORT) diagram. Patients and treatment characteristics are listed in Table 2. Histologically proven regional lymph nodes invasion (pN1) was 18 (14.6%). Bladder and rectum tumor invasion (pT4) was recorded in four (3.3%) patients. Detectable PSA level (> 0.2 ng/mL) was recorded in nine (7.3%) patients. Five-year LC, MFS, and OS were: 98.7%, 96.1%, and 95.1%, respectively. Actuarial 5-year and 10-year bDFS were 92.9% and 75.8%, respectively (Fig. 2). There was a significant difference between patients with GS ≤ 7 vs. GS > 7 (5-year bDFS: 95.5% vs 78.3 %; *p* = 0.001) (Table 3, Fig. 3). This difference maintained statistical significance (*p* = 0.014) even after Bonferroni’s correction for multiple comparisons. Grade 1–2 and Grade 3 acute GI toxicities were recorded in 56 (45.6%) and 3 (2.4%) patients, respectively. Grade 1–2 acute GU toxicities were recorded in 59 (48.0%) patients and Grade 3 GU toxicity in 4 (3.3%) patients, respectively. No patient had Grade 4 acute toxicity. Grade 1 and 2 late GI toxicities were recorded in 15 (12.2%) and 5 (4.1%) patients, respectively. No patient had Grade ≥ 3 GI toxicities. Five-year survival free from Grade 1 and Grade 2 GI toxicities were 87.0% and 96.7%, respectively. Grade 1, Grade 2, and Grade 3 late GU toxicities were recorded in 22 (17.9%), 16 (13.0%), and 5 (4.1%) patients, respectively. Five-year survival free from Grade 1, Grade 2, and Grade 3 GU toxicities were 78.6%, 88.6%, and 95.0%, respectively. No significant differences in terms of Grade ≥ 2 GU and GI toxicities were recorded based on dose to prostate bed, PNI, and adjuvant HT (data not shown).

**Table 2:** Patients and treatment characteristics

		No.	%
All patients		123	100
Age (median, range), years		64, 46–78	
pT			
	second	1	0.8
	2b	2	1.6
	2c	14	11.4
	3a	61	49.6
	3b	41	33.3
	4	4	3.3
PN			
	0	79	64.2
	1	18	14.6
	X	26	21.1
Surgical margins status			
	R0	51	41.5
	R1	72	58.5
Perineural infiltration			
	No	47	38.2
	Yes	76	61.8
PSA pre-surgery (median, range), µg/L		8.8, 0.4–55.0	
PSA post surgery (median, range), µg/L		0.06, 0.01–0.90	
Histopathologic grade, Gleason score			
	6	23	18.7
	7	69	56.1
	8–10	31	25.2
Lymphadenectomy			
	No	26	21.1
	Yes	97	78.9
Interval surgery-radiotherapy (median, range), months		4 (2–9)	
Radiotherapy dose to prostatic bed, Gy			
	64.8	18	14.6
	70.2	105	85.4
Prohylactic nodal irradiation			
	No	52	42.3
	Yes	71	57.7
Adjuvant hormone therapy			
	No	38	30.9
	Bicalutamide	48	39.0
	LH-RH analog	37	30.1

*N* number of patients; *PSA* prostate-specific antigen

**Table 3:** Impact of patient, tumors, and treatment parameters on 5 years biochemical disease-free survival (univariate analysis)

	No.	%	5-year bDFS (%)	*p*
Age, years				
≤65	72	58.5	92.0	0.166
>65	51	41.5	93.9	
pT				
2	17	13.8	92.3	0.515
3–4	106	86.2	93.2	
pN				
0	79	64.2	93.6	0.674
1	18	14.6	90.0	
X	26	21.1	91.3	
Margins status				
R0	51	41.5	93.1	0.441
R1	72	58.5	92.9	
Perineural infiltration				
No	47	38.2	93.9	0.115
Yes	76	61.8	92.6	
PSA pre-surgery, µg/L				
≤ 10	69	56.1	92.0	0.391
> 10	54	43.9	93.9	
PSA (post surgery), µg/L				
≤ 0.2	114	92.7	93.2	0.602
> 0.2	9	7.3	88.9	
Histopathologic grade, Gleason score				
≤ 7	92	74.8	95.5	**0.001**
8–10	31	25.2	85.5	
Lymphadenectomy				
No	26	21.1	91.3	0.499
Yes	97	78.9	93.3	
Radiotherapy dose to prostatic bed, Gy				
64.8	18	14.6	91.7	0.543
70.2	105	85.4	93.1	
Prophylactic nodal irradiation				
No	52	42.3	96.2	0.273
Yes	71	57.7	90.3	
Adjuvant hormone therapy				
No	38	30.9	91.3	0.486
Yes	85	69.1	93.7	
Adjuvant hormone therapy				
Bicalutamide	48	39.0	92.7	0.611
LH-RH analog	37	30.1	94.1	
Adjuvant hormone therapy				
Short-term (6 months)	23	27.1	100.0	0.183
Long-term (24 months)	62	72.9	91.8	

*bDFS* biochemical disease-free survival, *N* number of patients, *PSA* prostate-specific antigen

The bold entry was to emphasis the significant p valve

## Discussion

To our knowledge, this is the first prospective study suggesting the possibility to achieve higher bDFS rates by using a tailored treatment after RP for localized PCa. Despite poor prognostic features such as high rates of positive margins and perineural invasion, and inclusion of patients with pathologically involved pelvic nodes, our bDFS seems significantly higher (92.9%) compared with EORTC trial 22,911 [5] and other randomized trials with a biochemical recurrence rate of ~ 25% [3, 4]. Therefore, we could hypothesis that CIMA may improve patient outcomes by a combination of factors as discussed below. Obviously, this conclusion should be considered with caution, as (i) our study was a single arm trial, (ii) the apparent improvement of the results derives from a comparison with different studies. Therefore, we cannot rule out if the “Gleason grade migration” phenomena could have influenced on our comparisons result. In the EORTC 22,911 trial [9], for example, patients were enrolled between 1992 and 2001, clearly earlier compared with our study (2004–2009). Furthermore, comparing our experience with previous studies, we need to consider the RT technological evolution in recent years, which could have also influenced on the results. From the above-mentioned trial of Bolla et al. [9], RT was delivered with 2D technique, whereas in our study, 3D conformal technique was used.

When we analysed the three reported randomized studies [3–5] with radiation doses ranging from 60 to 64 Gy, most of our patients (85.4%) received a significantly higher dose (70.2 Gy) to the tumor bed. In addition, patients at risk of pelvic failures underwent PNI, which may be the reason of lower regional recurrences rate at this site, contrary to other studies. We believe that a combination of higher radiation doses with selective PNI may explain the comparable bDFS among patients with R0 vs. R1, and pN1 vs. pN0 disease, respectively. Furthermore, our results suggest the possibility to achieve an improved outcome after PNI compared with prostate irradiation alone as reported in other analyzes [14–16] in patients with metastatic pelvic nodes or high pelvic failure risk. It is noteworthy that, despite a higher tumor dose and selective PNI, the rate of acute and long-term toxicity was very low. Table 4 summarizes the results of randomized trials on postoperative RT in comparison with our series. Although, Table 4 clearly shows that the present study achieved a higher 5-year bDFS, we acknowledge the difficulties in comparing these results directly. In fact, our series has the highest rate of patients with lymph node metastases and high Gleason score, but at the same time the lower rate of R1 patients, whereas the proportion of patients with pre-surgical PSA > 10 ng/ml is poorly comparable and that of patients with post-surgical PSA > 0.2 ng/ml is similar to that of randomized trials.

**Table 4:** Results (biochemical disease-free survival): comparison with randomized studies

Study	Number of patients	Median follow-up	Adjuvant therapy	Proportion with					5-year bDFS
				pN1%	preop.PSA > 10 %	postop. PSA> 0.2%	R1%	Gleason score > 7%	
Thompson IM et al., 2006	214	10.6 and	RT: 60–64 Gy (2 Gy/fraction) to prostatic fossa and peri-prostatic tissue	0	52.7	35.2	90.0	19.0	NO
Wiegel T et al., 2009 (ARO 96-02 / AUO AP 09/95)	114	53.7 mo	RT: 60 Gy (2 Gy/fraction) to prostatic fossa and region of seminal vesicles with 1 cm margin	0	52.7	0	68.0	12.0	72.0
Bolla M et al., 2012 (EORTC 2911)	502	10.6 and	RT: 50 Gy (2 Gy/fraction) to prostatic fossa and region of seminal vesicles and peri-prostatic area + 10 Gy to prostatic fossa	0.4	NO	9.2	62.2	NO	74.0
Present series	123	67mo	RT: 64.8–70.2 Gy (1.8 Gy/fraction) to prostatic fossa and region of seminal vesicles with 1 cm margin ± PNI (45 Gy) ± HT	14.6	43.9	7.3	58.5	25.2	92.9

*bDFS* biochemical disease-free survival, *HT* hormone therapy, *mo* months, *NR* not reported, *PNI* prophylactic nodal irradiation, *preop* preoperative, *postop* postoperative, PSA prostate-specific antigen, *RT* radiotherapy *y* year

Other non-randomized studies using higher than standard dose ± HT and PNI were published [25–28]. Table 5 summarizes the results of these series compared with our trial. Although it is difficult to compare those series owing to heterogeneity in terms of margin status and pathological nodal stage, some of these studies seem to confirm that RT dose escalation in high-risk patients after RP may improve bDFS. Cozzarini and colleagues [25] reported 83.0% and 71.0% 5-year bDFS in patients receiving higher and lower than 70.2 Gy RT dose, respectively. This positive impact of dose escalation was also observed in R1 patients [27]. Furthermore, Ost et al. [25], prescribing a dose of 70–77 Gy, reported, 84% 7-year bDFS.

**Table 5:** Results (biochemical disease-free survival): comparison with non-randomized studies using high-dose radiotherapy

Study	Study design	Inclusion criteria	Adjuvant therapy	no. of pts	Median follow-up months	Proportion with					bDFS				
						pN1	Preop. PSA > 10	Postop. PSA> 0.2	R1	Gleason score > 7	All pts	N0	N1	R0	R1
Cozzarini C et al., 2009	Retrosp.	pT3-4 R0/1; pT2 R1; pN0	RT: 55.8–72 Gy to prostatic fossa ± HT	334	108	0.0%	NO	NO	66.0%	16.2%	83.0% ( ≥ 70.2 Gy) 71.0% (<70.2 Gy) (5-y)	NO	NO	96.0% ( ≥ 70.2 Gy) 81.0% (<70.2 Gy) (98 pts hormone-naive)	86.0% ( ≥ 70.2 Gy) 82.0% (<70. 2 Gy) (64 pts hormone-naive)
Bellavita R et al., 2012	Retrosp.	pT3-4 R0/1; any T R1; pN0	RT: 50–70 Gy (1.8–2 Gy/fraction) to prostatic fossa ± region of seminal vesicles (only pT3b) ± HT	182	55.6	0.0%	NO	30.0%	75.0%	43.5%	87.0% (3-y) 81.0% (5-y) 75.0% 10-y)	NO	NO	NO	NO
Ost P et al., 2012	Retrosp.	pT3-4 R0/1; any T R1; pN0	RT: 70–77 Gy (1.8–2.0 Gy/fraction) to prostatic fossa ± HT	225	60	0.0%	40.0%	NO	72.0%	20.0%	84.0% (7-y)	NO	NO	77.0%	86.0%
Katayama S et al., 2014	Phase II	pT3 R0/1; pT2 R1; pN + ; postop PSA recurrence; Roach nodal risk > 20.0% with inadequate nodal dissection (<10)	RT: 68 Gy (2 Gy/fraction) to prostatic fossa ± PNI 51 Gy (1.5 Gy/fraction) + HT	40	24	57.5%	NO	NO	NO	70.0%	90% (2-y)	NO	NO	NO	NO
Present series	Phase II	≤ 79 y; ECOG scale 0–2; pT2-4 N0-1 M0	RT: 64.8–70.2 Gy (1.8 Gy/fraction) to prostatic fossa + PNI 45 Gy ± HT	123	67	14.6%	56.1%	7.3%	58.5%	25.2%	92.9% (5-y)	93.6%	90.0%	93.1%	92.9%

*bDFS* biochemical disease-free survival, *preop* preoperative, *postop* postoperative, *y* years, *ECOG* Eastern Cooperative Oncology Group, *HT* hormone therapy, *NR* not reported, *PNI* prophylactic irradiation, *PSA* prostate-specific antigen, *pts* number of patients, *RT* radiotherapy, *Retrosp* retrospective

The influence of HT on bDFS after RP is difficult to assess owing to the variations in patients’ selection among retrospective studies [25–28]. We observed no significant effect of HT on bDFS in our study (5-year bDFS: 91.3% vs 93.7% in patients not receiving or receiving HT, respectively; *p* 0.486). We could hypothesize that this lack of advantage is due to HT prescription inhomogeneity. Indeed, the use of both androgen deprivation therapy and antiandrogen treatment (high-dose bicalutamide) may represent a limitation of our study. However, when CIMA trial was planned, the standard policy in our center was to inform patients on available evidence and different side-effects of both HTs and to let them choose. Furthermore, we did not observe any differences between the two HTs in terms of bDFS. Therefore, we postulated that the lack of response to HT may have been due to selective prescription and modulation based on risk factors, with HT prescribed only to higher risk subjects.

More generally, the advantages of combining postoperative RT with adjuvant HT after RP has been previously demonstrated. One study reported improved survival in patients with positive pelvic nodes who received both adjuvant HT and postoperative RT compared with patients receiving adjuvant HT alone [29]. Furthermore, a randomized trial showed that adjuvant systemic therapy based on high-dose bicalutamide may improve survival in patients treated with salvage RT after biochemical recurrence [30].

Despite the advantage of combining RT and HT in the adjuvant treatment of high-risk patients, the outcome of patients with high GS remains poorer. In our trial, a GS of 8–10 was correlated with lower bDFS and MFS compared with patients with GS 6–7. Other systemic therapies such as CT could be useful for these patients with higher risk of metastases. For example, in the setting of not-resected high-risk PCa, Fizazi et al. [31] reported a significant improvement of bDFS by combining CT to HT, compared with HT alone. Therefore, prospective trials to investigate the addition of CT to adjuvant treatment of high-risk PCa seem justified.

Unfortunately, being a single arm trial, our study is not able to provide information on the important problem of selecting patients for adjuvant therapies. However, we believe that our study has important clinical implications. Our results suggest that CIMA may improve bDFS by selective use of dose escalation, PNI, and adjuvant HT, with reasonable complications rates. Prospective clinical trials combining postoperative RT, adjuvant HT, and adjuvant CT to further reduce the risk of systemic relapses should be designed. These trials should be planned to enroll patients with high risk of systemic relapses, particularly patients with high GS.
